# Supplementary figures and images for: Pollen metabarcoding reveals broad and species-specific resource use by urban bees
Source: PeerJ. 2019 Feb 19;7:e5999. doi: 10.7717/peerj.5999 (PMC6385686; doi:10.7717/peerj.5999)

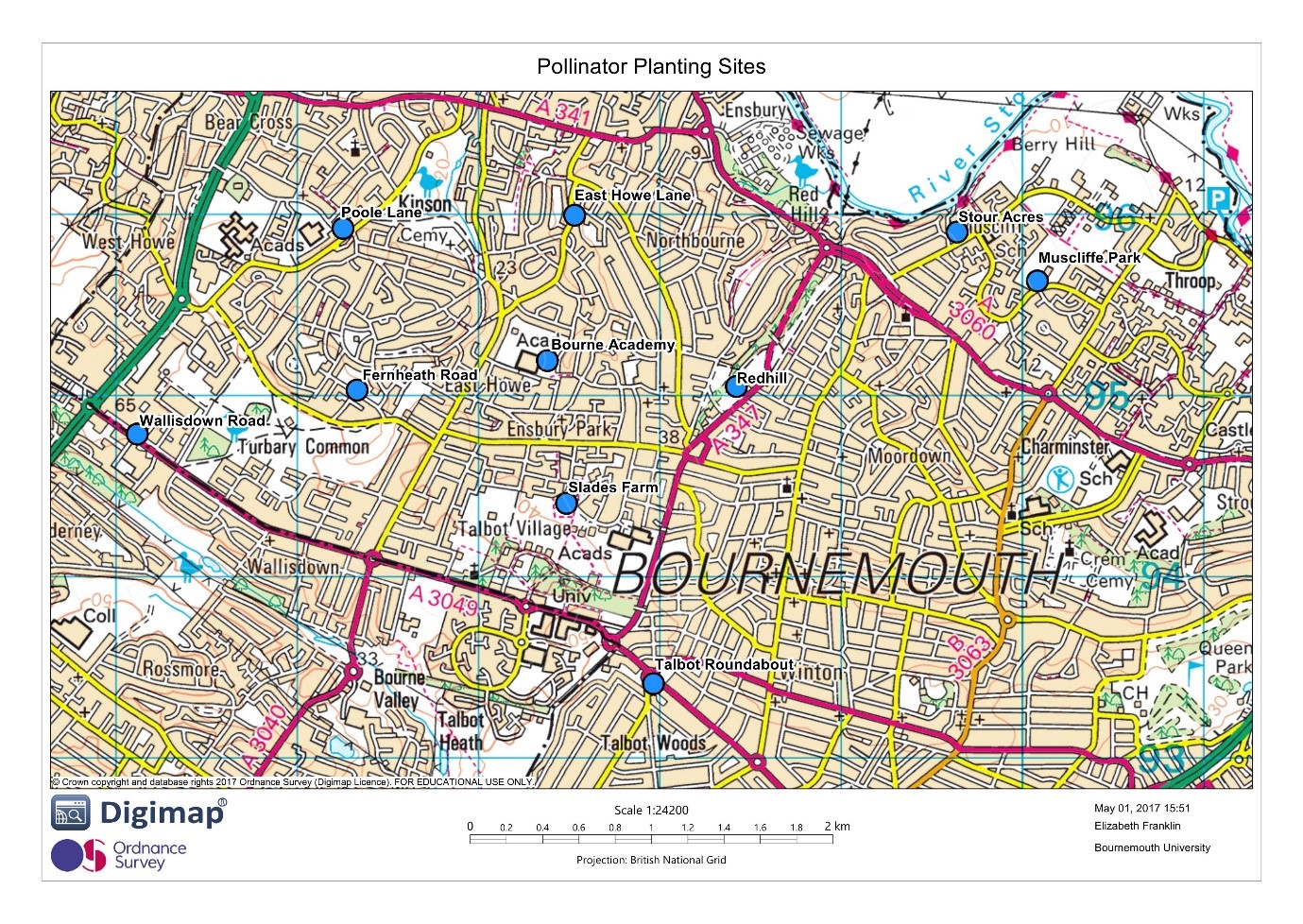

Supplement: Supplemental Information 1 [file peerj-07-5999-s001.jpg]
