# Supplementary material for: Pollen metabarcoding reveals broad and species-specific resource use by urban bees
Source: PeerJ. 2019 Feb 19;7:e5999. doi: 10.7717/peerj.5999 (PMC6385686; doi:10.7717/peerj.5999)
Supplement: Supplemental Information 5 — Both seed mixes contained a variety of wild seeds, with Papaver rhoas and Phacelia tanacetifolia being the dominant species by weight, followed by Rudbeckia, Leucanthemum and Chrysanthemum spp. Mixture 4 additionally contained Fagopyrum esculentum, Cichorium intybus and Helianthus annuus. Full details are contained in Table S2. [file peerj-07-5999-s005.docx]

| **Site Number** | **Site Name** | **Latitude, Longitude** | **Seed Mix** |
| --- | --- | --- | --- |
| **18** | Muscliffe Park | 50.760°N, 1.858°W | 3 |
| **21** | Talbot Roundabout | 50.741°N, 1.888°W | 4 |
| **22** | Slades Farm Bank | 50.749°N, 1.895°W | 4 |
| **23** | Redhill | 50.755°N, 1.882°W | 4 |
| **24** | Stour Acres | 50.726°N, 1.864°W | 4 |
| **28** | East Howe Lane | 50.763°N, 1.865°W | 4 |
| **29** | Bourne Academy | 50.756°N, 1.896°W | 4 |
| **30** | Fernheath Road | 50.762°N, 1.913°W | 3 |
| **31** | Poole Lane | 50.757°N, 1.912°W | 3 |
| **33** | Wallisdown Road | 50.753°N, 1.928°W | 3+4 |
