# Supplementary material for: Pollen metabarcoding reveals broad and species-specific resource use by urban bees
Source: PeerJ. 2019 Feb 19;7:e5999. doi: 10.7717/peerj.5999 (PMC6385686; doi:10.7717/peerj.5999)
Supplement: Supplemental Information 6 — The amount of each seed is expressed as percentage of total weight. Each mix contained three mixes from Moles Seeds: “summer picking mix” contained Zinnia , Calendula, Centaurea cyanus, Malope, Cosmos, Malva, Centaurea, Tagetes erecta, Aster and Gypsophila; “medieval carpet mix” contained Calendula, Linum rubrum, Malope, Centaurea, Echium, Borago and Nigella; and “express summer mix” contained Linum rubrum, Saponaria vaccaria, Malope, Papaver, Calendula, Nigella, Tagates, Pulsatilla, and Centaurea cyanus. Information was not available on the proportion of each seed in the three Moles Seeds mixes. [file peerj-07-5999-s006.docx]

|  | **Mixture** | |
| --- | --- | --- |
|  | **3** | **4** |
| ***Phacelia tanacetifolia*** | 7.6 | 7.1 |
| ***Fagopyrum esculentum*** | 0.0 | 2.4 |
| ***Cichorium intybus*** | 0.0 | 2.4 |
| ***Helianthus annuus*** | 0.0 | 2.4 |
| ***Rudbeckia* spp.** | 5.1 | 4.7 |
| ***Aster* spp.** | 0.3 | 0.3 |
| ***Carinatum* spp.** | 0.3 | 0.3 |
| ***Cosmos* spp.** | 0.3 | 0.2 |
| ***Leucanthemum vulgare*** | 6.4 | 5.9 |
| ***Anthemis arvensis*** | 0.3 | 0.4 |
| ***Centaurea cyanus*** | 1.1 | 1.1 |
| ***Chrysanthemum* spp.** | 2.9 | 2.7 |
| ***Papaver rhoas*** | 16.4 | 15.3 |
| ***Agrostemma gethago*** | 21.0 | 19.5 |
| **Summer Picking Mix** | 12.7 | 11.8 |
| **Medieval Carpet Mix** | 12.7 | 11.8 |
| **Express Summer Mix** | 12.7 | 11.8 |
