# Supplementary material for: Pollen metabarcoding reveals broad and species-specific resource use by urban bees
Source: PeerJ. 2019 Feb 19;7:e5999. doi: 10.7717/peerj.5999 (PMC6385686; doi:10.7717/peerj.5999)
Supplement: Supplemental Information 7 — Primers are shown in 5’ to 3’ orientation. [file peerj-07-5999-s007.docx]

| **Primer** | **‘Overhang’ sequence** | **Locus-specific primer** |
| --- | --- | --- |
| rbcLa-F | TCGTCGGCAGCGTCAGATGTGTATAAGAGACAG | ATGTCACCACAAACAGAGACTAAAGC |
| rbcLr506 | GTCTCGTGGGCTCGGAGATGTGTATAAGAGACAG | AGGGGACGACCATACTTGTTCA |
